# The Global Health Security index and Joint External Evaluation score for health preparedness are not correlated with countries' COVID-19 detection response time and mortality outcome

**DOI:** 10.1017/S0950268820002046

**Published:** 2020-09-07

**Authors:** Najmul Haider, Alexei Yavlinsky, Yu-Mei Chang, Mohammad Nayeem Hasan, Camilla Benfield, Abdinasir Yusuf Osman, Md. Jamal Uddin, Osman Dar, Francine Ntoumi, Alimuddin Zumla, Richard Kock

**Affiliations:** 1 The Royal Veterinary College, University of London, Hawkshead Lane, North Mymms, Hatfield, Hertfordshire, UK; 2 Institute of Health Informatics, University College London, London, UK; 3 Department of Statistics, Shahjalal University of Science and Technology, Sylhet 3114, Bangladesh; 4 Chatham House Centre on Global Health Security, Royal Institute of International Affairs, London, UK; 5 Fondation Congolaise pour la Recherche Médicale (FCRM), Brazzaville, Republic of Congo; 6 Institute for Tropical Medicine, University of Tübingen, Tübingen, Germany; 7 Department of Infection, Division of Infection and Immunity, UCL Centre for Clinical Microbiology, Royal Free Campus, London, UK; 8 NIHR Biomedical Research Centre, UCL Hospitals NHS Foundation Trust, London, UK

**Keywords:** COVID-19, GHS index, JEE, pandemic preparedness, risk analysis, surveillance system

## Abstract

Global Health Security Index (GHSI) and Joint External Evaluation (JEE) are two well-known health security and related capability indices. We hypothesised that countries with higher GHSI or JEE scores would have detected their first COVID-19 case earlier, and would experience lower mortality outcome compared to countries with lower scores. We evaluated the effectiveness of GHSI and JEE in predicting countries' COVID-19 detection response times and mortality outcome (deaths/million). We used two different outcomes for the evaluation: (i) detection response time, the duration of time to the first confirmed case detection (from 31st December 2019 to 20th February 2020 when every country's first case was linked to travel from China) and (ii) mortality outcome (deaths/million) until 11th March and 1st July 2020, respectively. We interpreted the detection response time alongside previously published relative risk of the importation of COVID-19 cases from China. We performed multiple linear regression and negative binomial regression analysis to evaluate how these indices predicted the actual outcome. The two indices, GHSI and JEE were strongly correlated (*r* = 0.82), indicating a good agreement between them. However, both GHSI (*r* = 0.31) and JEE (*r* = 0.37) had a poor correlation with countries' COVID-19–related mortality outcome. Higher risk of importation of COVID-19 from China for a given country was negatively correlated with the time taken to detect the first case in that country (adjusted *R*^2^ = 0.63–0.66), while the GHSI and JEE had minimal predictive value. In the negative binomial regression model, countries' mortality outcome was strongly predicted by the percentage of the population aged 65 and above (incidence rate ratio (IRR): 1.10 (95% confidence interval (CI): 1.01–1.21) while overall GHSI score (IRR: 1.01 (95% CI: 0.98–1.01)) and JEE (IRR: 0.99 (95% CI: 0.96–1.02)) were not significant predictors. GHSI and JEE had lower predictive value for detection response time and mortality outcome due to COVID-19. We suggest introduction of a population healthiness parameter, to address demographic and comorbidity vulnerabilities, and reappraisal of the ranking system and methods used to obtain the index based on experience gained from this pandemic.

## Introduction

On 31st December 2019, the World Health Organization (WHO) China country office was informed about a series of pneumonia cases with unknown aetiology in Wuhan city, Hubei Province [1]. By 15th July 2020, the disease, COVID-19, caused by infection with SARS-CoV-2 had infected 13 150 645 people and resulted in 574 464 deaths (4.4% reported case fatality ratio), affecting >200 countries/territories across the world [2]. Published mathematical models identified a number of countries in Asia, North America, Europe and Oceania with a higher risk of importation of the SARS-CoV-2 via infected people arriving from China [3–6]. On 22nd February 2020, Lebanon and Israel reported their first COVID-19 cases. No epidemiological link to China could be established through contact tracing, which suggested a link to an ongoing outbreak in Iran [7]. Before these reports, every country's first case had a history of travel to China.

The WHO declared COVID-19 as a pandemic on 11th March 2020 [2]. Earlier, the WHO had characterised COVID-19 as a Public Health Emergency of International Concern on 30th January 2020, their greatest concern being the potential for the virus to spread to countries with weaker health systems [2]. Thus, it was important to know how countries with different degrees of preparedness were responding to the pandemic, to inform epidemiological risk, and how resources could be best deployed and control measures applied in support of this global health emergency. Some countries were at a higher risk of importation of COVID-19 cases because of a higher volume of air passengers and travellers from China and understanding those countries' responses to this pandemic is also important [3, 4, 6]. These questions remain valid during all phases of the pandemic and especially during the process of removal of lockdown measures and opening of air bridges, when once again, risks of further spread increase. This is critical knowledge for what remains essentially a globally susceptible population, with few countries reporting immunity levels above an average of 5% in the community [8].

The Global Health Security Index (GHSI) is a comprehensive assessment and benchmarking of health security and related capabilities of 195 countries that make up the States Parties to the International Health Regulations. The GHSI is a project of the Nuclear Threat Initiative and the Johns Hopkins Center for Health Security, and was developed jointly with The Economist Intelligence Unit [9]. The GHSI provides an index of preparedness based on the capacity gaps of countries in their potential response to a pandemic, such as COVID-19, which most countries are ill-positioned to combat [9, 10]. The GHSI comprises six categories, 34 indicators and 85 sub-indicators based on 140 questions. Category 2 is the early detection and reporting of epidemics of potential international concern. We chose this category as our measure of the countries' reporting abilities. We further considered the risk of importation of COVID-19 from China to different countries based on air-flight passenger data [4]. Since most of the case reports prior to 20th February 2020 were linked to cases imported from China, we considered this approach a reasonable estimate of the relative risk of importing new cases into a given country. We also compared overall GHSI to mortality due to COVID-19 in the country (deaths per million), referred to as the mortality outcome hereinafter. The mean overall GHSI score is 40.2 out of 100. The high-income countries have an average score of 51.9 [9].

The Joint External Evaluation (JEE) is a voluntary, externally validated, collaborative assessment of 19 technical areas required to validate countries' capacities to prevent, detect and rapidly respond to public health risks [11]. Unlike the GHSI which is an academic tool developed to allow inter-country comparisons on pandemic preparedness, the JEE is a formal component of the WHO IHR Monitoring and Evaluation Framework which all UN member states are committed to implementing. The JEE is not designed for making inter-country comparisons but instead is a tool created to support WHO member countries in establishing a quantitative baseline assessment of IHR core capacities from which they can then measure their own progress over time. While the intention of JEE scoring has never been to draw inter-country comparisons, these have nonetheless occurred as politicians and national governments seek to assess their preparedness capacities against those of their neighbours or regional rivals. Ninety-six countries participated in the JEE scoring exercise and in this paper we use ReadyScore, which is the average of 19 technical areas included in JEE, as presented by Shahpar *et al*. [12]. This ReadyScore is calculated using either JEE 1.0 or JEE 2.0, depending on which assessment the country completed.

The objective of this study was to quantify and compare different countries' detection response times and mortality outcomes within specific dates, as related to the COVID-19 epidemic. Specifically, we evaluated the effectiveness of GHSI and JEE in predicting countries' COVID-19 detection response times as of 20th February 2020, and mortality outcomes as of 11th March and 1st July 2020, respectively.

## Methods

### The Global Health Security Index (GHSI) and Joint External Evaluation (JEE)

We used the mean value of GHSI Category 2 [9] – ‘early detection and reporting of epidemic of potential international concern’ – as an indicator of each country's preparedness for an epidemic/pandemic to evaluate a country's response to COVID-19. The countries are ranked between 0 and 100 (where 100 is fully prepared and 0 is not prepared at all). The countries with a score of 66.7 and above are categorised as ‘most prepared (MsP)’, 33.4–66.6 as ‘more prepared (MrP)’ and 0–33.3 as ‘least prepared (LeP)’ [9]. The mean overall GHSI score is 40.2 out of 100 and the USA lead the rank with 83.5 points. The high-income countries have an average score of 51.9. We also used JEE's ReadyScore to evaluate countries' responses to COVID-19 pandemic [12]. The score ranged from 0 to 100 and categorised as (i) Better Prepared (80–100), (ii) Work to do (40–79) and (iii) Not ready (0–39) [13]. The mean value of ReadyScore is 54.00 and Canada leads the ranking with an score of 93.00 [12, 13]. The details of these indicators are presented in the Supplementary material (Tables S1–S3). We used these overall GHSI scores to evaluate each country's preparedness and response to the pandemic and specifically to test associations using the detection response time and the related outcome, the deaths per million (death outcome at two different time points).

### Risk of COVID-19 importation

Haider *et al*. described the relative risk of importing COVID-19 cases from four major cities of China (Wuhan, Beijing, Shanghai and Guangzhou) to 168 countries and territories by considering the simulated air flight passenger data between 1st and 31st January 2020 [4]. The risk incorporated the probability of passengers being infected based on the outbreak sizes in the departure cities. Countries were grouped into four quartiles (Q1–Q4) based on the risk value (43 countries in the top quartile (Q4), 42 countries in the third quartile (Q3), 41 countries in the second quartile (Q2) and 42 countries in the bottom quartile (Q1), representing the lowest risk. We used this as a risk index of COVID-19 importation since it represented a proxy for the volume of travel of infected passengers from China into those countries and territories. Thus, countries with fewer passengers arriving from China had a lower risk of importation of COVID-19 cases.

We collected COVID-19 case and date of first report from WHO's daily situation updates until 20th February [14] as, during that period, most of the case reports outside of China were linked to the cases imported from China. On 22nd February, the WHO reported confirmed cases in Lebanon and Israel, both ultimately linked to Iran [7, 14]. The time lag between sampling a suspected case and reporting it at a country level has been reported to be up to 24–72 h [15]. Restricting our analysis until 20th February 2020 allowed us to investigate the cases linked to importation from China and enabled us to use the importation risk index based on the countries' air transportation links with China alone, as described above. Furthermore, we collected COVID-19 cases and death data from first reports until 11th March 2020 when WHO characterised COVID-19 as a pandemic and until 1st July 2020 from Worldometer [16].

### Statistical analysis

We collected further data mostly from United Nation's sources including country's population density [17], the percentage of the population aged 65 and above [18], human development index [19], gross domestic product (GDP) [20] and worldwide governance indicators (WGIs) [21].

For each of the preparedness categories, summary statistics are presented for the median [range] of the number of people infected with SARS-CoV-2 in the corresponding countries, and median [range] of the times from the beginning of the epidemic in China until detecting the corresponding first cases.

Kaplan–Meier curves were used to illustrate the time to first case detection from 31st December 2019. Countries without any case detected until 20th February 2020 were censored. Log-rank test was used to compare the rate of detection between the GHSI categories, and between the risk quartiles. Spearman's correlation was used to assess association between countries' responses to the COVID-19 epidemic and the GHSI, as well as the risk of COVID-19 importation. Multiple linear regression analysis was utilised to assess the amount of variation in either the time to detection of the first case that can be explained by the GHSI and JEE score and the importation risk among countries reporting cases by 20th February 2020. Since the values of importation risk were skewed, they were log transformed prior to further analysis. We performed multiple linear regression analysis for mortality outcome until 11th March 2020. Since the two indices, GHSI and JEE were strongly correlated (*r* = 0.82), we added them separately in the model. Finally, we performed negative binomial regression analysis to predict the impact of GHSI and JEE and other variables on countries' mortality outcome until 1st July 2020. Although negative binomial regression was preferred, there was not enough data for the period until 11th March 2020 to support the model and thus we ran multiple linear regression.

We have excluded China from the pre-pandemic (20th February 2020) analysis because we sought to evaluate countries' surveillance systems' effectiveness in detecting and monitoring the evolution of a potential pandemic and China is where the first cases were reported globally.

## Results

The median [range] value of GHSI score was 40.5 [3.7–98.2]: 22 countries in this study scored higher than the median value. As of 20th February, 26 countries reported COVID-19 cases imported from China: 13 were categorised as Most Prepared, 11 as More Prepared and 2 as Least Prepared countries in the GHSI. With respect to the risk of importation, 21 countries were in the top risk quartile (Q4), 4 countries in Q3, 1 country in Q2 and none in Q1.

### Time taken to identify the first case

As of 20th February, the countries in the top importation risk quartile (Q4) identified the first COVID-19 cases at median 28 [13–32] days after 31st December 2019, earlier than the countries in Q3, median 41 [32–51] days. Nepal was the only country in Q2 that identified its first COVID-19 case, 25 days after the start of the epidemic in China.

The MsP countries identified the first COVID-19 cases at median 26 [13–32] days after 31st December, earlier than the MrP countries at 30 [24–51] days and LeP countries at 27 [15–29] days.

**Fig. 1.**
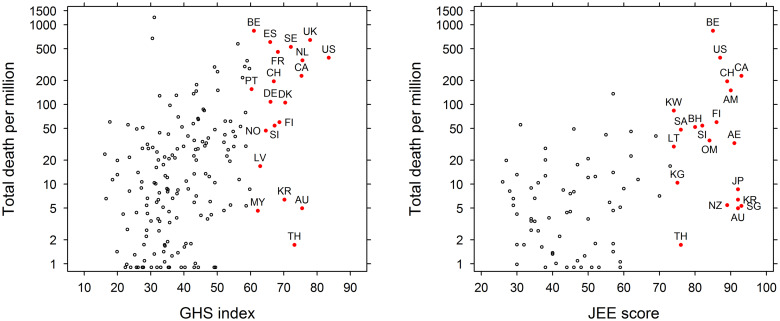
The Global Health Security Index (GHSI) overall score *vs*. the countries' mortality outcome due to COVID-19 (deaths/million) (left) and the JEE (ReadyScore) *vs*. countries' mortality rate due to COVID-19 (right). The countries with highest score in GHSI and JEE also had higher mortality rate due to COVID-19 (US, United States; UK, United Kingdom; NL, Netherlands; AU, Australia; CA, Canada; TH, Thailand; SE, Sweden; DK, Denmark; KR, South Korea; FI, Finland; SI, Slovenia; CH, Switzerland; DE, Germany; ES, Spain; FR, France; NO, Norway; LV, Latvia; MY, Malaysia; BE, Belgium; PT, Portugal; SG, Singapore; JP, Japan; AE, United Arab Emirates; AM, Armenia; NZ, New Zealand; OM, Oman; BH, Bahrain; SA, Saudi Arabia; KG, Kyrgystan; LT, Lithuania; KW, Kuwait).

The correlation coefficient between the risk of COVID-19 importation and the time to first reported COVID-19 case was −0.61, and the same coefficient between the risk of importation and the number of cases was 0.64. The correlation coefficient between the GHSI and the time to the first reported COVID-19 case was −0.32 (Fig. 1).

**Fig. 2.**
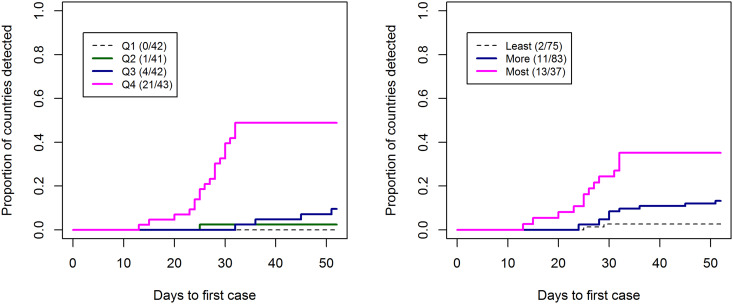
Kaplan–Meier curves for the time to first case detection from 31st December 2019 until 20th February 2020 stratified by (left) the risk of COVID-19 importation quartiles, (right) Global Health Security Indext (GHSI) categories (score: >66.6 as ‘most prepared (MsP)’, 33.4–66.6 as ‘more prepared (MrP)’ and 0–33.3 as ‘least prepared (LeP)’).

There were significant differences in the rates of first case detection between GHSI categories and between risk quartiles. Around 30% of countries in the highest GHSI score category had their first case detected by the end of January 2020, while 50% of countries in the top risk quartile (Q4) had their first detection by this time (Fig. 2). Nepal was identified as an outlier in the multiple linear regression analysis. If Nepal was excluded from the regression, the risk of importation alone explained the majority of the variation (*P* < 0.0001; adjusted *R*^2^: 0.63). However, the GHSI score had minimum impact in both cases. The patterns remained the same if Nepal was included in the analysis although the amount of variation explained was lower (adjusted *R*^2^: 0.33) (Table 1). When we used JEE's ReadyScore instead of GHSI, the model's predictive power remained very similar (adjusted *R*^2^: 0.66) with lower predictive value for JEE (Coefficient  = −0.05, *P* = 0.02) (Table 2).

**Table 1.** The linear regression and negative binomial regression models for COVID-19-related outcome and other explanatory variables including the GHSI

Duration	Pre-pandemic		Date of pandemic declaration		Post-pandemic declaration date	
Dates, until	20 February 2020		11 March 2020		1 July 2020	
Outcome variables	Duration of first case detection		Deaths per million		Deaths per million	
Model	Multiple linear regression		Multiple linear regression		Negative binomial regression	
	Coefficients	*P* value	Coefficients	*P* value	IRR (95% CI)	*P* value
Risk index	−2.97	<0.01	NA			
GHSI	−0.10	0.07	0.03	0.40	1.01 (1.02–1.09)	0.891
Total cases/million	NA		0.04	<0.01	1.01 (1.01–1.02)	<0.001
The percentage of the population aged 65 and above	NA		0.13	0.06	1.10 (1.03–1.16)	<0.001
WGIs	NA		−2.83	<0.01	1.12 (0.84–1.52)	0.023
GDP	NA		0.0001	0.31	0.99 (0.99–1.00)	0.585
Population density	NA		0.002	0.20	0.99 (0.98–0.99)	<0.01
	Adjusted *R*^2^: 0.63		Adjusted *R*^2^: 0.61		AIC: 1412	

For the period until 20 February 2020, multiple linear regression was performed, and the risk of importation of COVID-19 from China had higher predictive value than GHSI. For mortality outcome (deaths/million) until 1 July 2020, a negative binomial regression analysis was performed. The percentage of the population aged 65 and above were strongly associated with mortality rate. The incidence rate ratio (IRR) of 1.10 of the variable ‘the percentage of the population aged 65 and above’ indicates that an increase of 1% of population above 65 years of age increases the risk of death rate by 10%.

**Table 2.** The linear regression and negative binomial regression models for COVID-19-related outcome and other explanatory variables including JEE's ReadyScore [12, 13]

Duration	Pre-pandemic		Date of pandemic declaration		Post-pandemic declaration date	
Dates, until	20 February 2020		11 March 2020		1 July 2020	
Outcome variables	Duration of first case detection		Deaths per million		Deaths per million	
Model	Multiple linear regression		Multiple linear regression		Negative binomial regression	
	Coefficients	*P* value	Coefficients	*P* value	IRR (95% CI)	*P* value
Risk index	−2.93	<0.01	NA			
JEE (ReadyScore)	−0.06	0.02	−0.01	0.18	0.99 (0.98–0.99)	0.419
Total cases/million			0.04	<0.01	1. 01 (1.001–1.01)	<0.001
The percentage of the population aged 65 and above	NA		0.14	0.05	1.10 (1.01–1.21)	0.027
WGIs	NA		−2.62	<0.01	1.28 (0.82–1.96)	0.224
GDP (per capita)	NA		0.0001	0.21	0.99 (0.99–1.01)	0.971
Population density	NA		0.003	0.15	0.99 (0.98–0.99)	<0.001
	Adjusted *R*^2^: 0.66		Adjusted *R*^2^: 0.62		AIC: 619	

For the period until 20 February 2020, multiple linear regression was performed and the risk of importation of COVID-19 from China had higher predictive value than JEE. For deaths per million until 1st July 2020, a negative binomial regression analysis was performed. The percentage of the population aged 65 and above were strongly associated with mortality outcome. The incidence rate ratio (IRR) of 1.10 of the variable ‘the percentage of the population aged 65 and above’ indicates that an increase of 1% population above 65 years of age increases the risk of death rate by 10%.

### Mortality outcome due to COVID-19 (deaths/million)

The two indices, GHSI and JEE were strongly correlated (*r* = 0.82) indicating a good agreement between them. However, both GHSI (*r* = 0.31) and JEE (*r* = 0.37) had a poor correlation with countries' COVID-19-related mortality outcome. Among the 20 countries with highest mortality outcome, 10 countries also had the higher score in GHSI and five countries had higher score in JEE (mean ReadyScore) (Table S3 in the Supplementary material). The variables explaining the countries' mortality outcome are presented in Tables 1 and 2. For the period until the declaration of the pandemic (11th March 2020), the percentage of the population aged 65 and above was weakly positively correlated (Coefficient 0.13, *P* value = 0.06) but the GHSI was not a significant predictor (Table 1). When we used JEE in the model instead of GHSI, similar results were observed, with JEE remaining non-significant and the percentage of the population aged 65 and above as a significant variable (Coefficient 0.14, *P* value = 0.05) (Table 2).

In the negative binomial regression model, countries' mortality outcome was strongly predicted by the percentage of the population aged 65 and above (incidence rate ratio (IRR): 1.10 (95% confidence interval (CI): 1.01–1.21) while the overall score of GHSI (IRR: 1.01 (95% CI: 0.98–1.01) was not a significant predictor (Table 1). When we used JEE's ReadyScore in the model instead of GHSI, countries' mortality outcome were strongly predicted by the percentage of the population aged 65 and above (IRR: 1.10 (95% CI: 1.03–1.16)), while JEE (IRR: 0.99 (95% CI: 0.996–1.02) had little impact on the prediction (Table 2).

## Discussion

More than 200 countries/territories have been affected with COVID-19 resulting more than 11 million cases and 500 000 death as of 1st July 2020 [16]. We used datasets for three important time periods of the COVID-19 pandemic, including (i) 31st December 2019–20th February 2020 when every country's first COVID-19 case was linked to travel to China, (ii) 31st December 2019–11th March 2020 when the WHO declared the COVID-19 pandemic and (iii) 31st December 2019–1st July 2020, 6 months after the reporting of the first case in Wuhan, China. In these three periods of this epidemic, we evaluated two outcome variables including (i) time taken until first case detection and (ii) mortality outcome due to COVID-19 (deaths/million). However, in none of the models did GHSI or JEE status predict any expected outcome with confidence.

The first 26 countries reporting SARS-COV-2 are an important subset to study in order to understand how countries responded to this emerging disease. Until 20th February 2020, most of the COVID-19 case importations were linked to international travel from China. Our findings suggest that countries with a higher risk of importation of COVID-19, based on flight connectivity [4], detected cases earlier irrespective of their preparedness level, in contrast to the expectation that the higher GHSI scoring countries ought to more rapidly detect the presence of a novel pathogen in the population. China's notification to WHO and WHO's press briefing [22] encouraged by the ‘International Health Regulation 2005’ probably helped countries to proactively act on the risk [23, 24]. This implies that the risk of importation can be used as a proxy to account for the time it took COVID-19 to make an incursion into a particular country, after which most countries detected their first cases at speeds that were not affected by their GHSI or JEE preparedness scores. Our study further confirms that the health preparedness indices used either in the GHSI or JEE had low predictive value in terms of (i) number of cases detected in the country until 20th February 2020 when most cases were imported from China and (ii) mortality outcome (deaths/million) until either 11th March or 1st July 2020.

We do not know the exact dates when countries had their first imported cases, however, we inferred the risk of importing cases using direct and indirect air transportation links with China [4]. Data on the amount of technical assistance that the WHO and other institutions provided to less prepared countries are not readily available. However, our finding that countries with lower GHSI preparedness scores had comparable first case detection times to those that were deemed better prepared suggests that in many lower scoring countries, there is a good uptake of relevant guidelines and the local health staff are well-trained to respond to such outbreaks [25]. Although the capability of a country to perform large-scale testing will determine its long-term ability to detect COVID-19 cases reliably, and rapid first case detection is not the sole measure of success in containing an epidemic/pandemic, the latter can be seen as a good indicator of readiness and a more detailed investigation of the factors that determine its effectiveness is warranted.

Early case detection and accurate reporting of cases is essential for containing a pandemic so as to limit the spread of the disease both locally and globally. The current spread of COVID-19 outside China (as of 15th July 2020) is now driven mostly by community transmission and countries with higher preparedness scores are experiencing larger local outbreaks. For example, the USA has an overall GHSI of 92.1 (ranked 1st) and has identified more than 3.0 million cases as of 1st July 2020, of which the majority are now assumed to be locally acquired [16]. SARS-CoV-2 can apparently be transmitted without symptoms from a-/pre-symptomatic patients [26, 27] and super-spreading events may occur [28]. Countries that fail to detect cases in a timely manner run the risk of creating secondary outbreak foci [27]. Iran is one such example, with higher reported numbers of COVID-19-related deaths (*n* = 10 817) as of 1st July 2020. Additionally, it appears Iran has exported COVID-19 cases to at least 12 other countries including Bahrain, Kuwait and Lebanon that had not reported importing cases from China or other countries by the end of February 2020 [7]. A study by Tuite *et al*. estimated that there could be 18 300 (95% CI: 3770– 53 470) cases in Iran as of 24th February 2020 for it to export cases to those countries [7] while only 43 cases were reported on that day in Iran [2]. All of the above highlights the importance of having accurate pandemic preparedness metrics that would help to direct resources to where they are most needed in the event of another major outbreak. However, it seems that both the GHSI and JEE indicators do not correlate well with countries' ability to prevent or respond effectively to an epidemic.

Our findings showed that the countries with lower preparedness narrowed the gap of duration to detection of the first COVID-19 case. The countries with lower economic capacity especially those in South East Asia and sub-Saharan Africa are familiar with infectious disease epidemics. These countries, therefore, have extensive experience in early management of infectious diseases epidemics. For example, Vietnam, a country that has faced dengue [29] and chikungunya [30] epidemics in the last few years, has successfully controlled the COVID-19 epidemic in their territory and started laboratory preparedness prior to the first case being imported and reported in the country [16]. Vietnam had not reported any deaths even after 6 months [16]. The country took early measures to test, trace, isolate and quarantine suspected people and remains the only country of their size of population in the world without any mortality due to COVID-19 as of 1st July 2020 [31]. Investment in health infrastructure should reduce the risk of infection to a country and reduces overall risk of pandemic spread [32], but if not activated for political or other socioeconomic reasons, even well-structured capacity is inadequate in the face of a pandemic. Collective coordinated and comprehensive approaches that engage the entire machinery of government and international organisations including WHO should catalyse such preparedness in order to change the trajectory of the COVID-19 pandemic. Such coordinated efforts remain at the core of global health security.

The 10 countries worst affected with COVID-19 in terms of deaths per million are among the top 20 countries in terms of their overall GHSI scores. These scores are not correlated with the times taken to detect the first case in each country. Similarly, JEE's ReadyScore did not correlate well with detection of the first case in the country. For example, Vietnam ranked 50th (score: 49.1) and Sri Lanka 120th (score 33.9) within GHSI, and both countries responded well with less than one death per million (Vietnam reported no deaths). These rankings do not adequately weigh or consider the importance of universal health coverage, integration of national response services across sub-national jurisdictions and the critical importance of effective political leadership during times of crisis and such parameters are likely strongly correlated with time to detection and outcome, perhaps in part explaining the apparent paradox reported here. High income countries with a high GHSI or JEE may rely on professionals with competing interests and institutions with varying degrees of authority and responsibility across national and sub-national political boundaries to deal with a health crisis rather than taking individual and communal responsibility. Thus, the indicators and their respective weighting used in GHSI and the JEE might need to be radically revised in future from the lessons learned from the COVID-19 pandemic.

Our study has several limitations. First, the COVID-19 cases and death data reported from different countries might not be accurate for the total number of cases and deaths due to underreporting and inadequate testing, although early in the epidemic this was likely to be true in nearly all countries. There is a common case definition for COVID-19 globally [33], however, the testing and diagnostic capacity varies, which could affect both case and death counts. Going forward, the challenge will be to reduce such differences in reporting cases and deaths and the rate of detection is being improved generally. Second, the global pandemic awareness increases as a result of greater community engagement and this enables the governments to take the necessary steps to control the pandemic including acquiring and building the necessary diagnostic capacity. Therefore, the countries likely to import cases later than others had more time to prepare and were on greater alert. This might have affected the case detection speed in some countries. Our study has not incorporated this awareness factor in the model but it is unlikely to have been a major confounder especially as poorer countries are less able to disseminate information with lower media coverage and fewer consumers of electronic media. Third, the magnitude of the epidemic in China was growing during the study period, so the absolute risk of importation would have likewise grown with time for all of the countries. However, while the size of the epidemic increases the absolute importation risk, this would not change the relative risk values if the transportation links remained unchanged. On the other hand, travel bans were implemented in a number of countries, which would have affected the risk of importation, and which we could not address in our model. Nevertheless, given the relatively short time under consideration, we believe these limitations did not alter our findings substantially.

These global health preparedness indices (GHSI and JEE) are missing variables and inadequately weighting others that could more accurately capture the likely response of countries in such an emergency as COVID-19. As stated earlier, the WHO was more concerned with the risk of the pandemic to poor countries than rich ones at the beginning which has proven to be a very costly mistake, and the existence of the GHSI in its present form encouraged this position. One such missing variable could be a measure of vulnerabilities and/or dependencies on health systems from an aged population and from co-morbidities. Data are showing a high incidence of obesity, diabetes and cancers amongst fatalities, and the possible role of socioeconomic and environmental health risks from air pollution, relative poverty, poor housing and crowding, some of which have shown to be important drivers of the death rate due to COVID-19 in all countries [34]. The healthiness of any society including universal access to quality assured health services is another important measure of resilience as the ability to coordinate rapidly a societal response to a pandemic. A measure of trust in the political system or compliance with international health regulations may be important parameters to include in the index, as would be a One Health governance indicator showing potential for an integrated approach to prevention and response to emerging infectious diseases. This would devolve responsibility for public health to a wider disciplinary community, increasing the potential to prevent and respond to a pandemic. It would also improve the breadth of scientific advice to government on the one hand while also supporting a system of improved and more accountable governance of the actions arising from such advice on the other.

## Conclusion

The GHSI and JEE indices did not predict well countries' COVID-19 detection times and mortality outcome over the period of the study but it will be some months or years before a full assessment can be done. Countries with a higher risk of importation detected their first COVID-19 cases earlier, irrespective of their preparedness status as measured by the GHSI or JEE scores. Some limitations are inevitable in these analyses as all of the factors determining the speed of case detections could not be incorporated into our model. In the current COVID-19 pandemic emergency, countries with lower preparedness scores appear to have narrowed the gap of the time taken to detect first COVID-19 cases when compared to their so-called better-prepared counterparts. Long-term investment in health infrastructure for pandemic preparedness is essential but the true test of its efficacy is in a real pandemic. As shown by this study, especially early on in the pandemic of COVID-19, social and political factors and vulnerabilities not built into the GHSI and JEE evaluation can undermine effective action and these need to be addressed globally. We recommend the GHSI and JEE scoring tools be revised to include additional parameters that better estimate countries true pandemic preparedness and vulnerabilities, based on the lessons learned from the COVID-19 pandemic. Furthermore, the ongoing COVID-19 pandemic remains the most recent illustration of how global powers are ill prepared to lead health crises of international concern when their own societies are threatened, and the experience highlights the enduring need for greater political commitment to global health security and for a strengthening of the multilateral system to support a coordinated and effective response.

## Data Availability

All the data used in this article are publicly available. The full set of data we collected is available (on request to the corresponding author) for other researchers to assess and use for other research studies.

## Supplementary material

For supplementary material accompanying this paper visit http://dx.doi.org/10.1017/S0950268820002046. click here to view supplementary material
