# Supplementary material for: The Global Health Security index and Joint External Evaluation score for health preparedness are not correlated with countries' COVID-19 detection response time and mortality outcome
Source: Epidemiol Infect. 2020 Sep 7;148:e210. doi: 10.1017/S0950268820002046 (PMC7506172; doi:10.1017/S0950268820002046)
Supplement: Supplementary file 1 [file S0950268820002046sup001.docx]

**Supplementary document:**

**The** **Global Health Security index and Joint External Evaluation score for health preparedness are not correlated with countries’ COVID-19 detection response time and mortality outcome**

Najmul Haider^1*^, Alexei Yavlinsky^2^, Yu-Mei Chang^1^, Mohammad Nayeem Hasan^3^, Camilla Benfield^1^, Abdinasir Yusuf Osman^1^, Md. Jamal Uddin^3^, Osman Dar^4^, Francine Ntoumi^5,6^, Alimuddin Zumla^7,8^, Richard A Kock^1^

^1^The Royal Veterinary College, University of London, Hawkshead Lane, North Mymms, Hatfield, Hertfordshire

^2^Institute of Health Informatics, University College London, United Kingdom

^3^Department of Statistics, Shahjalal University of Science and Technology, Sylhet 3114, Bangladesh.

^4^Chatham House Centre on Global Health Security, Royal Institute of International Affairs, London, UK

^5^Fondation Congolaise pour la Recherche Médicale (FCRM), Brazzaville, Republic of Congo.

^6^Institute for Tropical Medicine, University of Tübingen, Germany.

^7^UCL Centre for Clinical Microbiology, Department of Infection, Division of Infection and Immunity, Royal Free campus,

^8^NIHR Biomedical Research Centre, UCL Hospitals NHS Foundation Trust, London, UK, London, United Kingdom

*****Corresponding Author

Table S1: The indicators used on GHSI

| The category |  |
| --- | --- |
| 1. Prevention | Prevention of the emergence or release of pathogens |
| 1. Detection and reporting | Early detection and reporting for epidemics of potential international concern |
| 1. Rapid response | Rapid response to and mitigation of the spread of an epidemic |
| 1. Health system | Sufficient and robust health system to treat the sick and protect health workers |
| 1. Compliance with international norms | Commitments to improving national capacity, financing plans to address gaps, and adhering to global norms |
| 1. Risk environment | Overall risk environment and country vulnerability to biological threats |
|  |  |

Table S2: The JEE technical areas ^1–3^

| **Element and technical areas** |
| --- |
| Prevention  1. National legislation, policy, and financing  2. IHR 2005 coordination, communication, and advocacy  3. Antimicrobial resistance  4. Zoonotic disease  5. Food safety  6. Biosafety and biosecurity  7. Immunization |
| Detection  8. National laboratory system  9. Real-time surveillance  10. Reporting  11. Workforce development |
| Response  12. Preparedness  13. Emergency Operations Centers  14. Linking public health and security authorities  15. Medical countermeasures and personnel deployment  16. Risk communication |
| Other hazards  17. Points of entry  18. Chemical events  19. Radiation emergencies |

**Table S3:** The top 20 countries with GHS overall scores and Joint External Evaluation (JEE) and countries with higher mortality (deaths/million) rate. Ten countries among the top-20 in overall GHSI also listed among the top-20 countries with higher mortality outcome (deaths/million). Five countries among the top-20 in JEE score also listed among the top-20 countries with higher mortality outcome (deaths/million).

| **Serial Number** | **Global Health Security Index^4^** | | **Joint External Evaluation ^25^** | | **Mortality rate due to COVID-19 as of July 15^th^ 2020 ^6^ (Countries above 1 M population)** | |
| --- | --- | --- | --- | --- | --- | --- |
|  | Country | Average Score | Country | Ready  Score | Country | Deaths/Million |
| 1 | **United States** | 83.5 | **Canada** | 93 | Belgium | 844 |
| 2 | **United Kingdom** | 77.9 | Singapore | 93 | United Kingdom | 664 |
| 3 | **Netherlands** | 75.6 | Australia | 92 | Spain | 608 |
| 4 | Australia | 75.5 | South Korea | 92 | Italy | 579 |
| 5 | **Canada** | 75.3 | Japan | 92 | Sweden | 549 |
| 6 | Thailand | 73.2 | United Arab Emirates | 91 | France | 461 |
| 7 | **Sweden** | 72.1 | **Armenia** | 90 | United States | 422 |
| 8 | Denmark | 70.4 | **Switzerland** | 89 | Peru | 376 |
| 9 | South Korea | 70.2 | New Zealand | 89 | Chile | 376 |
| 10 | Finland | 68.7 | **United States** | 87 | Netherlands | 358 |
| 11 | **France** | 68.2 | Finland | 86 | Ireland | 354 |
| 12 | Slovenia | 67.2 | **Belgium** | 85 | Brazil | 350 |
| 13 | **Switzerland** | 67 | Oman | 84 | Ecuador | 291 |
| 14 | Germany | 66 | Slovenia | 82 | Mexico | 282 |
| 15 | **Spain** | 65.9 | Bahrain | 80 | Canada | 233 |
| 16 | Norway | 64.6 | Thailand | 76 | Switzerland | 227 |
| 17 | Latvia | 62.9 | Saudi Arabia | 76 | Panama | 222 |
| 18 | Malaysia | 62.2 | Kyrgyzstan | 75 | Armenia | 200 |
| **19** | **Belgium** | 61 | Lithuania | 74 | North Macedonia | 189 |
| 20 | **Portugal** | 60.3 | Kuwait | 74 | Portugal | 165 |

References:

1 Bell E, Tappero JW, Ijaz K, *et al.* Joint External Evaluation—Development and Scale-Up of Global Multisectoral Health Capacity Evaluation Process. *Emerg Infect Dis* 2017; **23**: S33–9.

2 Shahpar C, Lee CT, Wilkason C, Buissonnière M, McClelland A, Frieden TR. Protecting the world from infectious disease threats: Now or never. BMJ Glob. Heal. 2019. DOI:10.1136/bmjgh-2019-001885.

3 World Health Organization (WHO). Joint External Evaluation ( JEE ). *Zoonotic Dis Action Packag Conf* 2017.

4 NTI and Johns Hopkins University Centre for Health Security. Global Health Security Index. 2019.

5 Prevent Epidemics: availabe at: https://preventepidemics.org/map/#. 2019.

6 Worldometer. Reported cases and deaths by country, territory, or conveyance: COVID-19 Pandemic. Worldometer. 2020. https://www.worldometers.info/coronavirus/#countries (accessed June 21, 2020).
